# Successful Management of Eagle Syndrome in a Patient With Repeated Office-Based, Ultrasound-Guided Nerve Blocks: A Case Report

**DOI:** 10.7759/cureus.102616

**Published:** 2026-01-30

**Authors:** Abdullah Nisar, Ali Sarfraz Siddiqui

**Affiliations:** 1 Anesthesiology, Aga Khan University, Karachi, PAK; 2 Anaesthesiology, Aga Khan University, Karachi, PAK

**Keywords:** eagle syndrome, glossopharyngeal neuralgia, pain management, peripheral nerves, ultrasound guided intervention

## Abstract

Eagle syndrome is a form of glossopharyngeal neuralgia, usually seen in older adults and characterized by pain in the throat, side of the face, and neck due to an elongated styloid process or calcified stylohyoid ligament. This condition can be managed with multimodal analgesia by multidisciplinary teams and may require surgical intervention for definitive relief. A 65-year-old female presented to the pain management clinic with chronic recurrent left-sided submandibular and neck pain. The pain was moderate in intensity (numeric rating scale (NRS) 5/10), episodic, sharp, burning, and electric shock-like in nature, and was associated with coughing and swallowing. She obtained only temporary and partial relief from medications. Her symptoms were successfully managed with repeated office-based ultrasound-guided glossopharyngeal nerve blocks using 3 ml of 0.25% ropivacaine and 4 mg dexamethasone. Significant improvement was maintained for approximately three months after the procedure.

## Introduction

Eagle syndrome is a rare type of glossopharyngeal neuralgia with an estimated incidence of 0.16% [1]. It is caused by elongation of the styloid process or calcification of the stylohyoid ligament, leading to irritation of adjacent cranial nerves and soft tissues [2]. Typically reported in the sixth decade of life with a high female predominance, it commonly presents as a unilateral throat, facial, or neck pain, and maybe associated with headaches, foreign body sensation, dysphagia, and pain exacerbated by swallowing or head movement, making it a diagnostic challenge in clinical practice due to symptom overlap with other orofacial pain syndromes [3]. Radiological evaluation, particularly a 3-D computed tomography (CT), is essential to confirm an elongated styloid process and define its anatomical relationship to surrounding structures [4]. Glossopharyngeal neuralgia is itself a rare pain condition that may be associated with Eagle syndrome when the elongated styloid process compresses or irritates the cranial nerve IX, producing lancinating or shock-like pain in its distribution [2]. While conservative pharmacologic measures, like anticonvulsants, are often employed first, many patients may experience only partial or transient relief, prolonging their symptom burden. In patients with recurrent or refractory symptoms, interventional modalities to target the glossopharyngeal nerve are now considered as the next step to pain management [5].

This case report describes a patient with recurrent glossopharyngeal neuralgia secondary to Eagle syndrome who was managed successfully with repeated office-based ultrasound-guided glossopharyngeal nerve blocks, illustrating a practical interventional option in the longitudinal management of this condition. The article was previously presented as a poster at the 24th AKU Annual Pain Symposium & 8th STSP Research Day at Aga Khan University, Karachi, on December 15, 2024, and atthe International Society of Pain and Neurosciences Conference, London, UK, on October 9, 2025.

## Case presentation

We present the case of a 65-year-old female patient who presented to the pain management clinic in 2014 with chronic, recurrent, left-sided submandibular and neck pain. The pain was episodic and associated with coughing and swallowing. She described it as sharp, burning, and electric shock-like, with a numeric rating scale (NRS) score of 5/10. She denied any history of trauma, neck surgery, or infection. Her medication history included gabapentin 300 mg and carbamazepine 200 mg taken thrice daily for the past year. The patient was switched to pregabalin 75 mg twice daily and continued on the same carbamazepine regimen. A diagnostic trigeminal nerve block was planned with an initial impression of trigeminal neuralgia.

The patient returned after eight months with persistent symptoms. She had undergone the diagnostic intervention without any relief. One year later, in 2015, she revisited the clinic with similar complaints. Neurology input was sought, and the diagnosis was revised to glossopharyngeal neuralgia. She subsequently underwent a fluoroscopic-guided glossopharyngeal nerve block with 2 ml of 0.25% ropivacaine and 40 mg methylprednisolone. Additional medications, including oxcarbazepine 300 mg twice daily, duloxetine 30 mg at night, and tramadol 50 mg twice daily, were prescribed.

Following this intervention, she experienced a symptom-free period of seven years and gradually tapered off her medications by herself. She had another relapse of pain in 2022, which was mild (NRS 3-4/10), burning in character, and associated with spicy food intake and cough. She was restarted on carbamazepine 200 mg once daily, duloxetine 30 mg at night, and tramadol 50 mg twice daily. Ultrasound-guided glossopharyngeal nerve blocks via the styloid process were performed in the clinic using ropivacaine and dexamethasone, with good effect.

In her most recent relapse, she again reported electric shock-like pain associated with food intake. She was prescribed duloxetine 30 mg at night and pregabalin 75 mg in the morning. A decision was made to administer repeated office-based ultrasound-guided glossopharyngeal nerve blocks, each with 3 ml of 0.25% ropivacaine and 4 mg dexamethasone. Three blocks were performed over 12 days, resulting in complete symptom relief (NRS 0/10). On her first follow-up after two weeks, she remained pain-free (NRS 0/10) and was advised to continue duloxetine 20 mg at night only. On subsequent follow-up after three months, the patient remained symptom-free.

## Discussion

Due to the rarity, patients with glossopharyngeal neuralgia are often misdiagnosed and mistreated as other, more common painful facial conditions, like trigeminal neuralgia [6]. However, less than 10% of cases have concurrent trigeminal neuralgia [7]. Pain occurs along the distribution of the glossopharyngeal and vagus nerves. It may radiate to involve the eye, nose, chin, or shoulder. Eagle’s syndrome may also be associated with vagal symptoms such as cough, hoarseness, syncope, and/or bradycardia, and seizures [8].

Eagle’s syndrome, first described by Dr. Watt Eagle in 1937, is a painful condition due to the elongated styloid process. It has a female predominance [2]. The elongation and ligament ossification can be due to endocrine disorders at menopause or end-stage renal disease. It can also be precipitated by trauma to the region [9].

Radiographic evidence is necessary to establish the diagnosis. While X-rays can be enough, CT imaging can help with precise anatomical relationships within the neck [2]. Carbamazepine or oxcarbazepine are considered the first-line therapy, with side effects being a major reason for their discontinuation [8]. Nonsteroidal anti-inflammatory drugs (NSAIDs) play a pivotal role in mitigating pain by modulating the local inflammatory process around the region [10]. Invasive options are considered when conservative therapy fails or is not tolerable. Surgical resection and shortening of the styloid process remains a definitive treatment but carries the risk of iatrogenic injury to surrounding vessels and nerves, poor cosmetic results, and a lengthy recovery period [2].

Nerve blocks have been a mainstay of treatment in chronic pain for patients for whom conservative therapies fail and cannot undergo surgery [11]. Ultrasound-guided techniques have recently found their way into mainstream practice due to the easy learning curve, real-time drug diffusion, low potential of adverse effects, and better tolerability. Regular access to chronic pain management care, particularly for women, has been a dilemma in low-and middle-income countries (LMIC) [12]. Socio-economic factors play a steering role in the lives of people belonging to LMIC, which explains our patient's regular loss to follow-up. Hence, it was decided to perform repeated office-based ultrasound-guided glossopharyngeal nerve blocks. A retrospective study from 2019 reported 83% effectiveness in their patients post single-shot, ultrasound-guided glossopharyngeal nerve block at the six-month follow-up [13]. While the block proved to be repeatable in this case report, a 2022 retrospective study also attests to this characteristic [14].

This case showcases the cost-effective and safe utility of ultrasound for office-based procedures. It also proved to be an effective and convenient option with a short operative time for patients belonging to LMIC.

## Conclusions

An older, adult female with chronic recurrent left-sided submandibular pain due to glossopharyngeal neuralgia from Eagle syndrome was successfully managed in a pain clinic with repeated office-based ultrasound-guided glossopharyngeal nerve blocks. This approach represents a safe, effective, and economical treatment option for patients in resource-limited settings.
